# Supplementary material for: Does the South-to-North Water Diversion Project promote the growth of enterprises above designated size in the water-receiving areas?—Evidence from 31 provincial-level administrative regions in China
Source: PLoS One. 2024 Feb 23;19(2):e0297566. doi: 10.1371/journal.pone.0297566 (PMC10889864; doi:10.1371/journal.pone.0297566)
Supplement: Attachment — Submitted filename: Response to Reviewers.docx [file pone.0297566.s002.docx]

Dear Editor and Reviewers,

Thank you for your valuable feedback and constructive comments on our manuscript titled *“Does the South-to-North Water Transfer Project Promote the Growth of Enterprises above Designated Size in the Water-receiving Areas?”* (Manuscript ID: PONE-D-23-14222).

Reviewers’ comments and our responses are as follows:

Reviewer #1:

1. The hypotheses developed should be mentioned directly after the text in literature review section, not in the introduction part.

Regarding your comment about the placement of the hypotheses, we have carefully considered your suggestion. Upon reviewing our manuscript, we agree that it would enhance the clarity and organization of our paper to directly mention the hypotheses after the literature review section, rather than in the introduction part. We have made the necessary revisions in the revised manuscript to address this point. Specifically, we have relocated the hypotheses immediately following the literature review section to ensure a more logical flow of information.

By making this adjustment, we believe that the revised manuscript now presents a more coherent structure and better aligns with the standards of academic writing in our field. We appreciate your insightful feedback, which has contributed to improving the overall quality of our manuscript.

2. In descriptive statistics standard deviation is so high, justify it.

In the modified manuscript, we have added a new paragraph in the revised manuscript to explain why we observed a high standard deviation in the descriptive statistics. We discuss the data sources and collection methods, and provide comparisons and explanations of relevant statistical indicators. Through these additional pieces of information, we aim to enhance the readers’ understanding of the rationality behind the data dispersion.

We believe that this explanation will help clarify and justify our data results, thereby improving the quality and comprehensibility of the paper. Thank you.

3. Conclusion should highlight the practical implication and theoretical contribution.

In the revised manuscript, we have made revisions to the conclusion section to highlight the practical significance and theoretical contributions. We have reorganized the paragraphs and explicitly mentioned the practical applications of our research and its contributions to theory. Specifically, we have emphasized the impact of our findings on decision-making in developing countries and the role of water management in economic development, as well as its implications for the advancement of theory in related fields.

Through these revisions, our aim is to make the conclusion section more clear and impactful, emphasizing the importance of practical applications and the contributions made to the advancement of knowledge in the academic domain.

We have carefully addressed all the comments and suggestions provided by the reviewer. The revised manuscript reflects these changes, and we believe that the modifications have significantly strengthened the overall quality and contribution of the study.

Once again, we express our gratitude to the editor and the reviewers for their time, expertise, and valuable input. We hope that our revised manuscript now meets the requirements for publication in your esteemed journal.

Sincerely,

Ting Wang

Communication University of China

Tingwang@cuc.edu.cn
